# The evaluation value of intracranial magnetic resonance angiography combined with carotid ultrasound in cerebral infarction

**DOI:** 10.12669/pjms.40.6.9312

**Published:** 2024-07

**Authors:** Xueliang Guo, Lin Sun

**Affiliations:** 1 Xueliang Guo Department of Neurology, Shengzhou People’s Hospital, The First Affiliated Hospital of Zhejiang University Shengzhou Branch, Shengzhou, Zhejiang Province 312400, P.R. China; 2 Lin Sun Department of Laboratory, Shengzhou People’s Hospital, The First Affiliated Hospital of Zhejiang University Shengzhou Branch, Shengzhou, Zhejiang Province 312400, P.R. China

**Keywords:** Intracranial, Magnetic resonance angiography, Carotid ultrasound, Cerebral infarction

## Abstract

**Objective:**

To explore the evaluation value of intracranial magnetic resonance angiography (MRA) combined with carotid ultrasound (CU) in patients with cerebral infarction (CI).

**Methods:**

A retrospective analysis was conducted on 122 patients with CI who underwent intracranial MRA combined with CU examination in Shengzhou People’s Hospital from January 2021 to October 2022. Vascular stenosis rate and CU parameters of patients with different degrees of nerve damage (ND) and size of CI lesion were analyzed.

**Results:**

The rate of vascular stenosis and ultrasound parameters significantly varied between patients with different degrees of ND and different sizes of CI lesion. Spearman test showed a significant positive correlation between vascular stenosis, pulsatile index (PI), and resistance index (RI) with the degree of ND and the size of CI lesions in patients. There was a significant negative correlation between peak systolic velocity (PSV) and end-diastolic velocity (EDV) and the degree of ND and the size of CI lesions (P<0.05).

**Conclusions:**

Intracranial MRA combined with CU can clarify the vascular stenosis and hemodynamic characteristics of patients with CI, and the combined approach closely correlates with the characteristics of CI, which can be used for disease assessment.

## INTRODUCTION

Cerebral infarction (CI), caused by acute interruption of cerebral arterial blood flow, is more prevalent in middle-aged and elderly populations and is associated with high rates of disability and mortality.^1^ Research shows that atherosclerosis and stenosis play an important role in the onset and progress of CI.^2^,^3^ Therefore, there is a need to improve methods for accurate evaluation of intracranial and cervical arterial anatomy and CI detection.^1^–^3^ Several imaging methods are currently used for CI diagnosis and assessment. Digital subtraction angiography (DSA) is considered the gold standard for the diagnosis of CI with high accuracy.^4^ However, DSA is costly and is associated with a certain degree of trauma, making it difficult to promote its application.^4^,^5^ Therefore, developing non-invasive CI diagnosis and assessment methods became a focus of research for decades.

Intracranial magnetic resonance angiography (MRA) is a non-invasive imaging method commonly used in the diagnosis and evaluation of CI.^6^ MRA has excellent correlation with cerebral angiography and can accurately show the number of diseased blood vessels, degree of stenosis, and specific location, as well as precisely reflect the degree of intracranial arteriosclerosis.^7^ Another method, carotid ultrasound (CU) is also an important non-invasive diagnostic technique for CI because of its simplicity and reproducibility, and is routinely used as a risk stratification tool for cerebral and cardiovascular events.^8^ CU can provide information on carotid intima-media thickness, stenosis nature and degree, evaluate blood flow velocity in the carotid artery and quantitatively evaluate arterial vascular function status using hemodynamic parameter information.^7^–^9^

Studies have showed that a combination of MRA and CU may be highly efficient in providing accurate anatomical information and identifying lesions in patients with carotid diseases.^10^,^11^ Most studies focus on the vascular stenosis rate and inter-reader agreement, but few on the correlation between vascular stenosis, CU blood flow parameters, and the degree of nerve damage (ND) and lesion size. This retrospective study aimed to assess the value and effectiveness of intracranial MRA combined with CU for evaluating the characteristics of CI in patients with different degrees of ND and size of CI lesion.

## METHODS

A total of 122 patients with CI who underwent intracranial MRA combined with CU examination in Shengzhou People’s Hospital from January 2021 to October 2022 were analyzed.

### Ethical Approval

This study was approved by the medical ethics committee of Shengzhou People’s Hospital (No.: 2021-046-01; Date: December 22, 2022). All procedures performed in study involving human participants were in accordance with the ethical standards of the institutional and/or national research committee(s) and with the Helsinki Declaration (as revised in 2013). The informed consent was waived by the ethics committee for the observational and retrospective nature.

### Inclusion criteria:

- Patients with confirmed acute CI, diagnosed through imaging examinations such as intracranial MRI.^12^
- Complete clinical data.
- First onset of acute CI.
- The time from onset to admission was less than 48 hours.

### Exclusion criteria:

- Patients with vascular diseases such as vascular dissection, aneurysm, and vasculitis, and those with a history of neck or intracranial vascular surgery.
- Patients with cardiogenic cerebral embolism such as atrial fibrillation or rheumatic heart disease.
- Patients with allergic constitution and a history of allergy to contrast agents.
- Patients with traumatic brain injury or intracranial hemorrhage.
- Patients with mental illness.
- Patients with malignant tumors.
- Patients with brain infection, or patients in the acute infection stage or other severe infections.
- Patients with aortic dissection or vasculitis.

### Intracranial MRA examination

**Fig.1:**
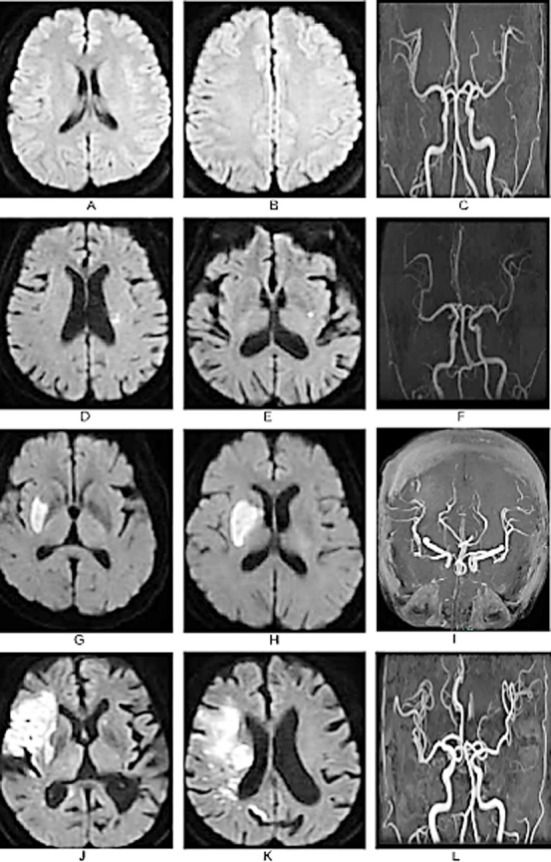
Intracranial MRA imaging of patients with different infarct sizes. A-C: No abnormalities were found in the head MRA imaging and blood vessels of normal healthy individuals; D-F: Head MRA of patients with small area cerebral infarction, small acute infarction in the left basal ganglia area, mild stenosis of the right middle cerebral artery; G-I: Head MRA of patients with moderate area cerebral infarction, moderate acute cerebral infarction in the right basal ganglia, and moderate stenosis of the right middle cerebral artery; J-L: Head MRA of patients with extensive cerebral infarction, extensive cerebral infarction in the right basal ganglia, and severe stenosis of the right internal carotid artery.

The procedure was done using the GE Optima MR360 1.5 T MRA scanner (GE Healthcare, Beijing, China). Vertebral artery bifurcation to the top of the skull was scanned using a fast 3D FLASH sequence. Parameters were set as follows: Scanning time, 218 seconds; Field of view, 11 cm; Matrix, 220 mm×220 mm; Layer thickness, 1.2-1.4 mm; Reverse angle, 70^º^; TE, 6.6 ms; TR, 19.5 ms; and Pixel size, 1 mm×1.07 mm ×0.78 mm. The degree of stenosis of diseased blood vessels was evaluated based on the MRA images (Fig.1): grade 0, normal; grade 1, complete visualization of distal vessels with a stenosis degree ≤ 50%; grade 2, distal vascular imaging with a stenosis degree>50%; grade 3, occlusion and no visualization of distal blood vessels. Grade 1-3 were considered as vascular stenosis.^13^

### CU examination

**Fig.2:**
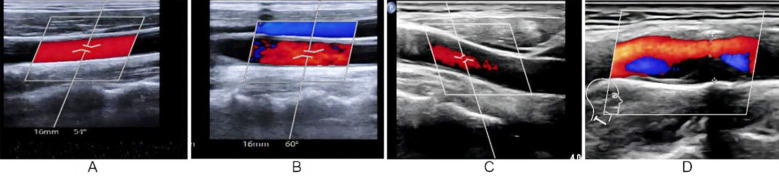
Neck vascular ultrasound of patients with different infarct sizes. A: Color Doppler ultrasound of normal and healthy neck blood vessels, no stenosis observed; B: Mild stenosis of the neck blood vessels can be seen on color Doppler ultrasound in patients with small area cerebral infarction; C: Moderate stenosis of the neck blood vessels can be seen on color Doppler ultrasound in patients with moderate area cerebral infarction; D: Severe stenosis of the neck blood vessels can be seen on color Doppler ultrasound in patients with extensive cerebral infarction.

Siemens ACUSON S2000™ (Siemens, Munich, Germany) ultrasound diagnostic equipment was selected for inspection. The probe frequency was set to 5-8 MHz. Patient was lying flat with a soft pillow under the neck, and the tested area of the neck was exposed. Patient was guided to turn their head to one side. Contralateral examination was performed, starting from the anterior edge of the sternocleidomastoid muscle. Multi section and multi angle scanning on the distal, middle, proximal, and internal carotid arteries and their branches of the common carotid artery were performed. Plaque condition and the degree of vascular stenosis in the carotid artery were measured. Subsequently, an inspection on the other side was performed. The ultrasound diagnostic device was placed at the bifurcation of the carotid artery to perform carotid blood flow parameter detection. The average values were calculated three times, including peak systolic velocity (PSV), end diastolic velocity (EDV), pulsatile index (PI), and resistance index (RI) of the internal carotid artery (Fig.2).

### Collected data

- Baseline data: gender, age, alcohol consumption, smoking status, body mass index (BMI), history of hyperlipidemia, history of diabetes, history of hypertension, and results of intracranial MRA combined with CU examination.
- Degree of ND: it was evaluated using the National Institutes of Health Stroke Scale (NIHSS) scoring scale. The scoring range was 0-42 points, with higher scores indicating more severe ND. NIHSS score <15 was considered mild, 15-30 as moderate, and>30 as severe.^14^
- Size of the CI lesion: it was categorized into small infarction (maximum diameter of 1.6-3.0cm), medium infarction (less than one lobe or maximum diameter of 3.1-5.0cm), and large infarction (larger than one lobe of the brain, or with a maximum diameter exceeding 5cm).^15^

### Statistical Analysis

SPSS version 20 (IBM Corp, Armonk, NY, USA) was used for analysis. Continuous variables were reported as mean and standard deviation. One-way analysis of variance (ANOVA) was used to evaluate the statistical significance of continuous variable differences between different degrees of ND and different lesion sizes. Bonferroni post hoc test was used for paired comparison. Categorical variables were reported as frequency and percentage, and Chi-square tests were used to evaluate the differences between the groups, different degrees of ND, and different lesion sizes. Spearman test was used to analyze the correlation between vascular stenosis, PSV, EDV, PI, RI, and the degree of ND and lesion size in patients. A *p*-value less than 0.05 was considered statistically significant. All reported *p*-values were bilateral.

## RESULTS

A total of 122 patients (69 males and 53 females) with CI were included in the analysis. The general information of the patients is shown in Table-I. The levels of vascular stenosis, PI, and RI in patients with mild to moderate ND or small to moderate lesions were significantly lower than in patients with severe or large lesions, and the lowest levels were observed in patients with mild or small lesions (*P*<0.05). PSV and EDV were significantly higher in patients with severe or large lesions, and the highest levels was observed in cases of mild or small lesions (*P*<0.05) (Table-II).

**Table-I:** General information of patients

General Information		Mean±standard deviation or n (%)
Male	/	69 (56.56)
Age (Years)	/	63.48±8.61
Time from onset to hospitalization (Hours)	/	14.72±7.46
Degree of ND	Mild ND	33 (27.05)
	Moderate ND	62 (50.82)
	Severe ND	27 (22.13)
Size of CI lesions	Small lesions	31 (25.41)
	Moderate lesions	65 (53.28)
	Large lesions	6 (4.92)
Hyperlipidemia (Yes)	/	59 (48.36)
Diabetes (Yes)	/	39 (31.97)
Hypertension (Yes)	/	69 (56.56)

***Note:*** ND=nerve damage; CI=cerebral infarction.

**Table-II:** Baseline characteristics.

Characteristic	n	Vascular stenosis[n(%)]	PSV (cm/s)	EDV (cm/s)	PI	RI
Mild ND	33	17 (51.52)	80.79±16.96	54.70±11.56	0.96±0.19	0.78±0.13
Moderate ND	62	45 (72.58)^a^	65.89±14.21^a^	42.95±10.41^a^	1.18±0.19^a^	0.98±0.17^a^
Severe ND	27	26 (96.30)^ab^	48.15±10.57^ab^	25.78±5.33^ab^	1.35±0.21^ab^	1.06±0.18^ab^
*χ*^2^/F		14.827	38.553	63.820	30.644	25.744
*p*-value		0.001	<0.001	<0.001	<0.001	<0.001
Small lesions	31	14 (45.16)	79.81±17.76	52.06±9.99	0.93±0.17	0.81±0.13
Moderate lesions	65	48 (73.85)^c^	66.52±14.64^c^	44.35±12.46^c^	1.19±0.18^c^	0.96±0.19^c^
Large lesions	26	26 (100.00)^cd^	48.19±10.78^cd^	25.65±5.40^cd^	1.36±0.21^cd^	1.06±0.18^cd^
*χ*^2^/F		21.358	32.331	45.454	39.894	15.585
*p*-value		<0.001	<0.001	<0.001	<0.001	<0.001

***Note:*** Compared with mild cases,

a P<0.05; Compared with moderate, ^b^P<0.05; Compared with small lesions,

c P<0.05; Compared with medium lesions, ^d^P<0.05. ND = nerve damage; S = second; PSV = peak systolic velocity; EDV = end diastolic velocity; PI = pulsatile index; RI = resistance index.

Spearman test confirmed a significant positive correlation between vascular stenosis, PI, and RI and the degree of ND and the size of CI lesions in patients (*P*<0.05). There was a significant negative correlation between PSV, EDV, and the degree of ND and the size of CI lesions (*P*<0.05) (Table-III).

**Table-III:** Analysis of the correlation between the combined MRA/CU and characteristics of CI.

Item		Vascular stenosis	PSV	EDV	PI	RI
Degree of ND	*r*-value	0.348	-0.634	-0.728	0.559	0.519
	*p*-value	<0.001	<0.001	<0.001	<0.001	<0.001
Size of CI lesion	*r*-value	0.418	-0.593	-0.558	0.613	0.439
	*p*-value	<0.001	<0.001	<0.001	<0.001	<0.001

***Note:*** ND = nerve damage; CI =cerebral infarction; PSV = peak systolic velocity; EDV = end diastolic velocity; PI = pulsatile index; RI = resistance index.

## DISCUSSION

The study indicated that intracranial MRA combined with CU have high application value in the diagnosis of CI, and can effectively clarify vascular stenosis and hemodynamic characteristics of patients with CI. Wang et al.^16^ found that MRA examination can effectively characterize cerebral hemodynamic status in patients with cerebral ischemia. MRA allows to image arteries that are unreachable by a catheter approach, and the acquired images can be used to create specific cross-sectional views in the direction of choice. This method does not use radiation, is non-invasive and have demonstrated a strong reproducibility and high resolution for soft tissues. This allows to clearly observe changes in the outer wall and lumen of blood vessels, and to present the distribution of atherosclerotic plaques, determine their size, and accurately evaluate the condition of carotid artery stenosis.^16^,^17^98 normal subjects and 106 TIA patients who underwent MRI examination within 72 h after the last symptom onset including the DWI sequence to exclude acute cerebral infarction were enrolled. The blood flow of the cranial total, the area of the internal carotid artery and vertebral artery, the average velocity, and the average blood flow were obtained and compared in normal subjects and TIA group. Analysis of Variance (ANOVA Kim et al.^18^ confirmed that MRA examination can effectively identify vascular stenosis in patients with acute CI, guide clinical targeted treatment, reduce the risk of symptomatic intracranial hemorrhage, and ensure favorable prognosis and outcome. Jaiswal et al.^19^ found that MRA can accurately evaluate the condition of vascular stenosis. Diagnostic sensitivity, specificity, positive predictive value, and negative predictive value of MRA for anterior cerebral artery stenosis in patients with CI was 85.9%, 90.0%, 98.2%, and 50.00%, respectively. Similarly, diagnostic sensitivity, specificity, positive predictive value, and negative predictive value for posterior cerebral artery stenosis were 73.5%, 86.7%, 96.2%, and 40.0%, respectively. The above is consistent with the conclusions of our study.

However, Chen et al.^20^ reported that there is still a certain risk of missed diagnosis and misdiagnosis when using MRA for diagnostics and evaluation of acute CI. It can be hypothesized that the accuracy of MRA as a diagnostic tool may be affected by the operator’s skills. Additionally, plaque calcification at the examination site may also lead to unclear MRA imaging, thus affecting the examination results. These shortcomings may be resolved by combining MRA with other inspection methods. Zhao et al.^21^ showed that ultrasonic examination can improve the diagnostic efficacy of acute CI with high sensitivity and specificity and can evaluate the stability of atherosclerotic plaque. Yin et al.^22^ demonstrated that CU can dynamically monitor atherosclerotic plaque, reflect changes in vascular wall, and determine the extent of lesions by measuring indexes such as EDV, PSV, etc. These observations are consistent with the results of our study. Our results also showed that patients with different characteristics (degree of DN, size of CI lesion) had different degrees of vascular stenosis which significantly correlated with the changes in PSV, EDV, PI, and RI. Our results suggested that combined MRA and CU examination can clarify the severity of the patient’s condition, help guide clinical implementation of targeted treatment, and avoid improper or excessive treatment.

Psychogios et al.^23^ pointed out that the main clinical manifestation of carotid artery stenosis is abnormal hemodynamic changes in the internal carotid artery. When the stenosis of the carotid artery occurs, arterial RI and PI increase abnormally, normal blood circulation in the body is obstructed, and the brain experiences varying degrees of ischemia and hypoperfusion, resulting in a slowdown in blood flow velocity.^24^ There is a significant correlation between the severity of the stenosis and the changes in the measured indexes. The more severe the carotid artery stenosis, the lower the EDV and PSV, and the higher the RI and PI are.^9^,^23^ CU clearly identifies the thickening of the intima media, plaque location, and volume of blood vessels, and can accurately measure the hemodynamic status of the carotid artery, providing a reference basis for evaluating the degree of carotid artery stenosis.^25^ Therefore, MRA combined with CU can comprehensively examine cerebral blood vessels and cervical arteries, comprehensively evaluate the patient’s condition, and avoid missed diagnosis or misdiagnosis.^26^

### Limitations

First, it is a single-center retrospective study, which may be subject to selection bias due to incomplete medical records. Second, neither group was randomly assigned, and baseline information may be imbalanced and biased. The rate of vascular stenosis and ultrasound indicators may be influenced by human or technical factors. Finally, the prognostic evaluation value of the combined MRA/CU examination method was not analyzed. Further higher quality research is needed to validate our conclusions.

## CONCLUSION

Intracranial MRA combined with CU can clarify the vascular stenosis and hemodynamic characteristics of patients with CI, and the combined approach closely correlates with the characteristics of CI, which can be used for disease assessment.

### Authors’ contributions:

**XG** conceived and designed the study.

**XG** **and** **LS** collected the data and performed the analysis.

**XG** was involved in the writing of the manuscript and is responsible for the integrity of the study.

All authors have read and approved the final manuscript.
